# Hepatic pseudoaneurysm after traumatic liver injury; is CT follow-up warranted?

**DOI:** 10.1186/1752-2897-8-18

**Published:** 2014-11-14

**Authors:** Lene Østerballe, Frederik Helgstrand, Thomas Axelsen, Jens Hillingsø, Lars Bo Svendsen

**Affiliations:** Department of Surgery and Liver Transplantation C, University Hospital of Copenhagen, Blegdamsvej 9, 2100 Copenhagen, Denmark; Department of Radiology, University Hospital of Copenhagen, Rigshospitalet, Blegdamsvej 9, 2100 Copenhagen, Denmark

## Abstract

**Introduction:**

Hepatic pseudoaneurysm (HPA) is a rare complication after liver trauma, yet it is potentially fatal, as it can lead to sudden severe haemorrhage. The risk of developing posttraumatic HPA is one of the arguments for performing follow-up CT of patients with liver injuries. The aim of this study was to investigate the occurrence of HPA post liver trauma.

**Methods:**

A retrospective study from 2000-2010 of conservatively treated patients with blunt liver trauma was performed to investigate the incidence and nature of HPA. After the initial CT scan patients were admitted to the department and if not clinically indicated prior a follow-up CT was performed on day 4-5.

**Results:**

A total of 259 non-operatively managed patients with liver injury were reviewed. 188 had a follow-up CT or US and in 7 patients a HPA was diagnosed. All aneurysms were treated with angiographic embolization and there were no treatment failures. There was no correlation between the severity of the liver injury and development of HPA. 5 out of 7 patients were asymptomatic and would have been discharged without treatment if the protocol did not include a default follow-up CT.

**Conclusions:**

In conclusion, this study shows that HPA is not correlated to the severity of liver injury and it develops in 4% of patients after traumatic liver injury. In order to avoid potentially life-threatening haemorrhage from a post trauma hepatic pseudoaneurysm, it seems appropriate to do follow-up CT as part of the conservative management of blunt and penetrating liver injuries.

## Introduction

A hepatic pseudoaneurysm (HPA) is an unusual but potentially lethal complication after blunt or penetrating liver injury [1–4]. A pseudoaneurysm is a false aneurysm that develops from a leakage of an injured artery into the surrounding tissues forming a cavity outside the artery. It can be distinguished from a haematoma as it continues to communicate with the artery resulting in a high-pressure cavity with the risk of rupture [5]. A pseudoaneurysm can develop anywhere in relation to an injured artery, but common sites are hepatic or splenic artery branches following trauma [6].

Development of a HPA is mainly described in patients after liver trauma but is also reported after hepato-biliary surgery, pancreatitis, gallstone disease and liver-related invasive procedures such as liver biopsies [5, 7–9]. The diagnosis of an HPA is made with either arteriography, contrast-enhanced computer tomography (CT-angiography) or Doppler Ultrasound (US) [5].

Symptoms of an HPA may vary from clinically silent to signs of rupture with intra-peritoneal haemorrhage or rupture into the gastrointestinal tract, venous, portal or biliary system [2, 4, 7]. Previous studies have found that the risk of developing an HPA after liver trauma is 1,2-6,1% [4, 10, 11]. The evidence for follow-up radiology after non-operatively managed liver trauma to identify HPA remains debatable [10–14].

The aim of the present work was to investigate the incidence and outcome of HPA in non-operatively managed patients suffering from liver injury after trauma.

## Methods

This retrospective study included all patients conservatively managed after liver injury admitted to the Trauma Centre at the University hospital of Copenhagen, Rigshospitalet. The study period was from January 2000 to December 2010. Indication for conservative treatment was hemodynamic stability after initial resuscitation and no signs of peritonitis or extravasation of hollow viscous [15]. The exclusion criterion was patients operated for other intra-abdominal injuries. Patients managed primarily with angiographic embolization were also excluded. The follow up period was from admission to hospital until discharge. The patients all received 3 days of antibiotics with a cephalosporin. The conservatively managed patients were observed in the trauma ICU ward for the first 24 hours after admission and if hemodynamically stable, referred to the surgical ward for further observation. All included patients had an initial CT, followed by a control CT or US after 4–5 days. Children and patients with minor injuries might have had an UL instead in order to detect increasing amount of free fluid. Two different non-blinded radiologists evaluated all initial and follow-up CT scans. Ultrasound findings were not re-evaluated by another radiologist. Liver injuries were graded according to the scale of the American Association for the Surgery of Trauma Organ Scaling Committee (AAST) [16].

Outcome was development of HPA on the follow up scan after conservatively managed liver trauma. Patient demographics, mechanism of injury, hospital course, blood pressure and heart rate on presentation, findings on CT/US, blood transfusions within the first 48 hours and interventions were abstracted from the medical files. Continuous data are presented as medians with 25th and 75th interquartile range (IQR). Categorical data are reported as proportions. Correlation between grade of liver injury and risk for HPA was calculated by Mann Whitney U test. P <0.05 was regarded significant.

The study was registered at http://www.clinicaltrials.gov. No: NCT01938885.

## Results

**Figure 1:**
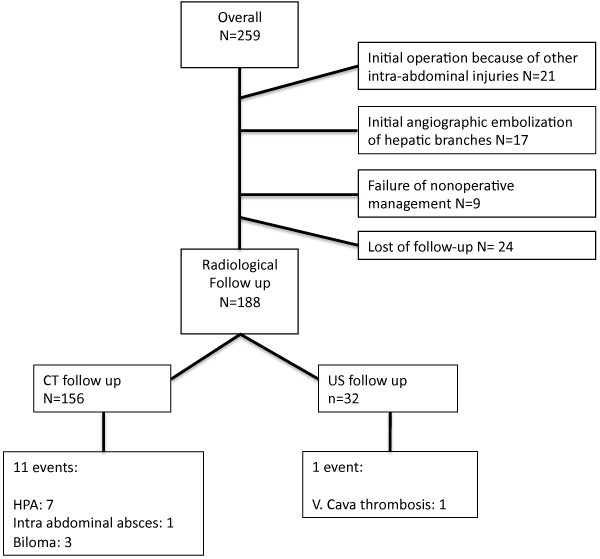
**Flow-chart of the population.** Detailed*:* Conservatively managed liver trauma patients grouped according to follow-up radiology and events on follow-up scans.

In a total of 259 patients, the liver injury was initially managed conservatively. 47 patients were excluded due to active management with either laparotomy or angioembolization prior to protocol CT on day 4–5. 24 patients were lost to radiological follow-up. Figure 1 shows the study flow chart. 188 patients were included and 156 patients had a CT undertaken at median of 5 days (4–5 days) and 32 patients a US at median of 5 days (3–6 days). Children and adolescents under 18 years constitute 28% (N = 52) of the study population, of whom 18 patients (35%) had a US follow up. Patient characteristics of the included patients are shown in Table 1. US was preferably done in children to investigate the amounts of free fluid, no one in this group showed clinical or US signs of delayed bleeding and only one complication was detected (Figure 1).

**Table 1:** **Demographics and pre-hospital data for the 188 patients who were followed-up with either CT or US**

Total number of patients		188
Age, median (IQR)		24 (16–35)
Male, n (%)		102 (54)
*Comorbidity, n (%)		40 (21)
Blunt trauma, n (%)		184 (98)
Liver grade (AAST)		
	I, n (%)	19 (10)
	II, n (%)	80 (43)
	III, n (%)	68 (36)
	IV, n (%)	20 (11)
	V, n (%)	0
	No data, n (%)	1 (0,5)
Systolic blood pressure <90 mmHg, n (%)		14(7)
Pulse rate >100 bts/min, n (%)		51(27)
Blood transfusion, n (%)		28 (15)
**mortality, n (%)		1 (0,5)

*comorbidity defined as medication-dependent other disease.

**Overall mortallity (n = 1) was caused by cerebral trauma.

n (%) = number of patients (percentage of total, n =188).

The follow-up CT revealed HPA in 7 patients (Table 2). Thus the overall incidence of HPA 5 days after liver injury was 4%. Two patients with HPA presented with symptoms of hemodynamic failure with the need of blood transfusion, the other 5 patients had no symptoms. Three patients with HPA had a low-grade liver injury (grade II) and the remaining four patients sustained high-grade liver injuries (Table 2). There was no correlation between grade of liver injury and risk for HPA (p = 0.277). All seven patients with HPA were managed with angiographic embolization and discharged without complications.

**Table 2:** **Characteristics of the 7 patients who developed HPA post liver trauma**

Patients with HPA	Gender	Age	Mechanism of trauma	Liver injury grade*	Symptoms of HPA	Blood transfusion
1	Female	25	Blunt	2	No	No
2	Male	20	Blunt	2	Yes	Yes
3	Male	45	Blunt	2	No	No
4	Male	9	Blunt	3	No	No
5	Male	30	Blunt	3	Yes	Yes
6	Female	42	Blunt	4	No	No
7	Male	42	Blunt	4	No	No

*Detailed:* 2 patients presented with symptoms of hemodynamic failure with hypotension, decreasing haemoglobin and abdominal pain. *No correlation between grade of liver injury and risk for HPA (p = 0.277).

## Discussion

In the present retrospective study including 188 patients with radiological follow-up, we found that 4% developed HPA within 5 days after the liver injury. There was no correlation between the risk for HPA and the grade of liver injury.

This is among the largest studies performed regarding follow-up radiology of conservatively managed patients with a focus on HPA, post-liver trauma. Our default CT/US follow-up rate was 87% (N = 24 lost to follow up). Earlier studies report of follow-up rates of 49% [12], 51% [11], and 60% [17]. Most of the existing knowledge about HPA post liver trauma is from case-reports with patients presenting with clinical symptoms [2, 3, 7, 18–22]. A few larger retrospective studies show incidences of HPA post liver trauma between 1,2%-6,1% similar to our data [4, 10, 11]. In general, the studies are heterogeneous and with varying follow-up rates and strategies. Two of them look at liver trauma patients initially operated [4, 10].

Cox et al. did the largest study of routine follow-up of 530 conservatively managed liver trauma patients [23]. They found 3 patients with the need of intervention. Their conclusion is that routine follow-up is unnecessary. Pachter et al. did a multicenter study, where they followed-up 198 out of 404 conservatively managed liver trauma patients and did not report any HPA [12]. They found 14 (3,5%) patients with delayed haemorrhage managed either with blood transfusion, angio-embolization or operation. Whereas the patients came from 13 different trauma centres with variables of conservative management, follow-up rate and time to follow-up, they were unable to reach definitive conclusions.

An HPA after hepatic injury can lead to fatal outcome because of sudden severe haemorrhage. As an HPA might be ‘silent’ until enlargement and rupture, the diagnosis is difficult when no default follow up CT is performed. In our study most patients with HPA were asymptomatic, which is also found in earlier studies [3, 10, 11]. There was no correlation between symptoms of HPA and degree of liver injury (Table 2). However, the number of patients with HPA is too small to draw any conclusions.

In agreement with the literature, we did prophylactic angiographic embolization of the HPA [2, 3, 5]. Several studies have shown that traumatic pseudoaneurysms on the splenic artery may thrombose spontaneously [11, 24–27]. It is uncertain whether an HPA follows the same benign course. No proven methods exist to determine whether an HPA will thrombose or rupture, and we do not really understand the natural progression of HPA. Further conservative observation in our study might have contributed to the understanding of the nature of an HPA, but we did not find it ethically correct to further observe the patient with a risk of severe consequences.

We mainly did CT follow-up instead of US. Few studies have compared CT with US [5, 28] and follow-up radiology remains an ongoing debate [14, 17, 29–32]. The efficacy of Doppler US to rule out HPA is not to date sufficiently illuminated. CT is gold standard, but CT also increases exposure of radiation, which is of special concern in children and pregnant women [33, 34].

Surprisingly, three out of seven patients with low-grade liver injuries (grade II) developed HPA. Croce et al. found that two out of six patients initially operated with grade II injuries developed HPA [4]. Furthermore, from case studies, we know that minor invasive procedures such as liver biopsy may cause HPA [9]; this indicates that even a minor liver injury might initiate HPA development. Thus, follow-up radiology of both low and high-grade liver injuries seems appropriate.

Our follow-up period was median of 5 days, however the duration of follow-up, hospital admission and how to monitor the conservatively managed patients is debatable [35] and there exists no data on the risk of developing HPA later than 5 days after liver injury.

Limitations of this study include the retrospective aspect and lack of CT follow up in 24 patients. Furthermore, 32 patients were followed-up with US, which might have missed the detection of an HPA in these patients.

Future studies comparing US with CT in follow up of liver trauma are still warranted. Doppler US might equal CT follow up [3]. In addition, studies to further illuminate the nature of HPA are lacking. Still, we need to know more about spontaneous thrombosis of HPA and look into methods of follow up patients without repeating potentially harmful investigations. We did not rapport complications after angioembolization, whereas earlier studies have shown morbidity rates of 6-20% [36, 37].

## Conclusions

In conclusion, this study shows that HPA is not correlated to the size of liver injury and it develops in 4% of patients after traumatic liver injury. In total ¾ of patients with HPA were asymptomatic. In order not to miss this complication, we have focused on early detection and definitive treatment before enlargement and rupture. Based on our results we suggest early detection with follow-up CT during the primary admission and definitive treatment before enlargement and rupture.
